# Mimicking Schizophrenia: Reducing P300b by Minimally Fragmenting Healthy Participants’ Selves Using Immersive Virtual Reality Embodiment

**DOI:** 10.3389/fnhum.2018.00504

**Published:** 2019-01-04

**Authors:** Bernhard Spanlang, Birgit Nierula, Maud Haffar, J. Bruno Debruille

**Affiliations:** ^1^Event-Lab, Department of Clinical Psychology and Psychobiology, Universitat de Barcelona, Barcelona, Spain; ^2^Institut d’Investigacions Biomèdiques August Pi i Sunyer (IDIBAPS), Barcelona, Spain; ^3^Department of Neuroscience, McGill University, Montreal, QC, Canada; ^4^Douglas Hospital Research Center, Montreal, QC, Canada; ^5^Department of Psychiatry, McGill University, Montreal, QC, Canada

**Keywords:** schizophrenia, self, P300b, P3b, LPC, LPP, functional significance, embodiment in immersive virtual reality

## Abstract

The most robust and clear biological index differentiating persons with schizophrenia from healthy controls is the drastic reduction of the amplitude of their P300b event-related brain potential (ERP). However, the cause of that reduction remains obscure. Nevertheless, the P300b belongs to the family of the late posterior positivities (LPPs) which are closely related to the consciousness of the meaning of the stimulus in the task *for the participants themselves (*e.g., the: *I* am seeing the target stimulus for which *I* have to respond*)*. The fragmentation of the self present in schizophrenia, could thus be the cause. If this were true, then P300bs should be somewhat reduced in healthy participants when their self representations are temporarily and minimally fragmented. We tested this hypothesis by using the innocuous fragmentation of the self that occurs in virtual reality (VR). There, participants can have a fragment of their self in an avatar they feel embodied in, within a VR room, while having another fragment of their self in their real body in the real room where they know they are. Our participants were thus equipped with a head mounted display in which they viewed a virtual room where a female humanoid avatar was facing them. She was lifting her right hand in synchrony with the participants, in order to induce in them a feeling of embodiment. Stimuli were a frequent green- and a rare red-disk, the oddball stimulus, occurring over the right hand of the avatar. Participants had to perform a Go/NoGo task, lifting their right hand to the frequent green disk and repressing this action for the oddball red disk. In the syncMove block of trials the avatar was lifting her right hand synchronously with the participant, disturbing her self representation as confirmed by the debriefing session. In the noMove block, the avatar remained immobile. In the classic block, only the red and the green disk were displayed on a monochrome background, neither the room nor the avatar were shown. As predicted, P300bs were found to be smaller in the syncMove block than in the noMove- and the classic-block in participants who had the classically large P300b oddball effect between ERPs to the frequent and those to the rare stimuli. Reduced P300bs of schizophrenia could thus be partly due to self fragmentation. Results may also open an avenue of research to the functional significance of LPPs and the content of the consciousness indexed by these potentials.

## Introduction

Only 7 years after the first description of the P300b event-related brain potential (ERP; Sutton et al., 1965), it has been shown that its voltage (or amplitude) is drastically reduced in schizophrenia patients relative to healthy controls (Roth and Cannon, 1972), even more so when their symptoms are severe (Mathalon et al., 2000), with partial normalization when treatment is efficient to curtail these symptoms (Coburn et al., 1998). Dozens of studies allowed to conclude that this remarkable reduction is the most robust biological difference between healthy and schizophrenia people (for a review see Ford, 1999), far beyond anatomical differences, genes and electrophysiological indexes other than the P300, such as measures of sensory gating (Ritsner, 2009). P300bs are also found to be reduced in many other pathologies (for a brief review, see Picton, 1992), but to a much lesser extent.

The P300b is generally accepted as indexing the consciousness of the meaning of the stimulus for the subject in the cognitive task at hand (Kutas et al., 1977; Donchin and Coles, 1988; Vogel et al., 1998). Nevertheless, despite the fact that their P300bs have often less than half of the amplitude of those of average healthy persons, patients do not appear to suffer from a severe lack of consciousness of the meaning of the stimulus. The radical reduction of their P300b appears out of proportion with their behavioral deficits in the task. These deficits mainly consist in delayed responses (Nuechterlein, 1977; Vinogradov et al., 1998) and thus not in a gross lack of the awareness that a target stimulus just occurred. Together with the much more modest, but nevertheless sizable, P300b reductions found in pathologies other than schizophrenia (Picton, 1992), this surprising phenomenon points to P300b factors besides the conscious appraisal of the meaning of the stimulus for the task.

One, quite trivial, factor that is common to the diverse pathologies where reduced P300bs are found (Picton, 1992) is that the people who are participating to these studies know they are contributing as patients rather than as healthy controls. Their awareness of being sick arguably corresponds to some kind of diminished representation of their body, which is part of the representation of the self and of the self concept (Skaff and Pearlin, 1992). This, usually overlooked, factor could be relevant here precisely because the most important P300b reductions are found in schizophrenia patients, in whom the representation of the self is arguably the most affected.

The word “schizophrenia” itself was coined by Bleuler in 1908 precisely because it means split mind (Ashok et al., 2012) and thus split self. Patients with schizophrenia report being inhabited by the spirits of others. Many say that they hear voices and thoughts of other persons in their mind. Further, their sense of agency is perturbed (Jeannerod, 2009) for example, after doing something, they occasionally report that they did it not knowing why or because the voices ordered it (Hacker et al., 2008). Some of them even attempt (or commit) suicide upon these orders (Chadwick and Birchwood, 1994). Embodiment can also be disturbed, as some patients report feeling as if they were within the body of others (Nordgaard et al., 2017)^1^. It is for these people that psychoanalysts developed the concept of the fragmentation of the self (Hamm et al., 2017). In any case, the representations they have of themselves is notably diminished, as it does not include an important part of their own mental activity, which is attributed to, and thus bound with, representations of other entities, such as those of aliens, gods, voices, spirits etc.

The P300b factor besides the consciousness of the stimulus meaning mentioned above could be self-representations. In effect, conscious perception appears to be linked to the self. For instance, when we perceive a stimulus, we are also, and at the same time, conscious that it is *us*, who are perceiving it (Thompson and Varela, 2001; Baars et al., 2003). This means that the representations that are activated by the stimulus are automatically bound to the representations of the self. These stimulus-activated representations include those of the meaning of the stimulus in the task at hand (e.g., representations that this is the target stimulus for which a response is required). The more fragmented the representation of the self, the greater the chances the stimulus will be bound to a small fragment. The drastic reduction of schizophrenia patients’ P300bs might thus be due a binding that links the stimulus to only a small fragment of their self. Considering self-representations could thus open an avenue of research as to the functional significance of reduced P300bs in schizophrenia (as in Ebisch and Gallese, 2015). Changes in the self-representations could be reflected in the P300b. If this were the case, temporarily (and minimally) fragmenting the self representations of healthy participants should reduce their P300s.

To test this hypothesis, we used embodiment in immersive virtual reality (EIVR) because this technology has the potential to induce a fragmentation of the self. There, in effect, participants have a fragment of their self in the avatar they feel embodied in, within the VR, while having another fragment in their real body in the real room where they know they are. This fragmentation of the self is innocuous and minimal, most likely because participants know that these two fragments are part of a whole. Namely, they are part of the experience of VR while being in a real room. Participants can, therefore, bind all the elements of the situation. Incidentally, it has to be noted that this contrasts with pathological fragmentation, such as the schizophrenia one. When the thought of an unknown other bursts into the patients’ thinking, it is total surprise. It does not correspond to anything known for patients. So there is no frame, no prior representation, in which they can include themselves together with this unknown other.

To achieve our goal, we thus choose a setting where an avatar was facing participants in the room shown by the head mounted display they were equipped with. A sensor was attached to the right hand of each participant who was asked to lift it for the frequent stimulus and to prevent that movement for the oddball stimulus. In the critical condition, the avatar copied without noticeable delay the right hand movement of the participants, which is why this condition was called the syncMove one. This synchrony of movement has been shown to evoke the illusion of being embodied in avatars (Sanchez-Vives et al., 2010; Slater et al., 2010). In order to focus the attention of the participant on this latter movement, the rare- and the frequent-stimulus that the P300b classical oddball protocol had to include were occurring only a few centimeters above the wrist of the avatar.

As mentioned, compared to healthy controls, schizophrenia patients have, on average, much smaller P300b. This is observed despite the fact that both the degree to which they can be fragmented and the way they are so immensely differ across them. Some have voices, some do not, some feel the presence of a stranger within their mind, some feel such a presence outside their body, just next to them, some feel that they are Jesus while still acknowledging their real identity etc. EIVR is a different way of fragmenting the self but this technically induced experience follows the same principles as patients’ experiences. Therefore, if fragmentation of the self is the cause of the smaller P300b amplitudes observed in patients, it should be reduced by EIVR in our healthy participants.

## Materials and Methods

### Participants

Thirty-one right-handed female participants were recruited among students of the University of Barcelona. Four participants were excluded as they did not have clear P300b effects, that is, there was no clearly larger P300bs to the oddball-NoGo trials than to the frequent-Go trials. This led to a final sample size of 27 participants with an average age of 20.9 years (ranging from 19 to 33). They reported not suffering from any major psychological or neurological problem and had normal or corrected-to-normal vision. This study was carried out in accordance with the recommendations of the Tri-Council Policy Statement: Ethical Conduct for Research Involving Humans (TCPS, Canada) and approved by the Research and Ethics Board of the Department of Psychology of the University of Barcelona, with written informed consent from all subjects, who gave it in accordance with the Declaration of Helsinki. The protocol was approved by the Research and Ethics Board of the Douglas Mental Health University Institute and the Ethics Board of the Department of Psychology of the University of Barcelona.

### Stimuli and Procedure

**Figure 1:**
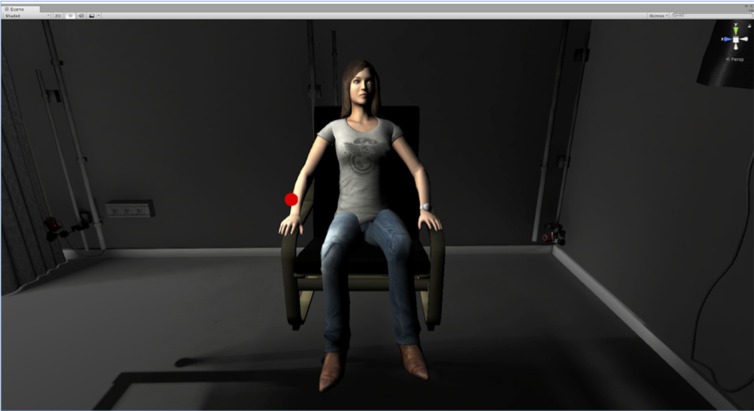
This image shows the virtual room in which participants were immersed with the head mounted display and the avatar that was facing them in this room in both the syncMove and the noMove condition. It also shows the stimulus-disk that was turning either red (noGo oddball), green (Go, frequent) or black (for inaction). In the classic condition, there was no room and no avatar, just darkness and the disk turning green, red or black, which had to be responded as in the syncMove and the noMove condition.

After the electrode cap was placed on their scalp, participants were equipped with an Oculus DK2 head mounted display which has built in head-orientation tracking-capabilities. They could then see a room where an avatar was facing them (see Figure 1). An inertial measurement unit (IMU) sensor, the wired Trivisio Colibri Inertial Motion Tracker (Trivisio), was placed on their right hand and connected to the VR display computer *via* the VR peripheral network (VRPN; Taylor II et al., 2001) so that the avatar right hand was moving upward when that of the participant was. Unity3D was used in combination with embodiment and physiologic measurement software as described in Spanlang et al. (2014).

Participants first had to get used to these movements. Then, they were given the task instructions. These instructions informed them that there were three different virtual environments in each of the three blocks of trials used. In two of these environments, there was an avatar, that is, a 3D picture of a woman sitting in front of them, as depicted in Figure 1. She had a black disk hovering over her right hand, which was flashing either red or green. In the third environment, only the disk appeared. There was no woman and no room, just a dark background. In all conditions, the participants viewed the scenario from a 3rd person perspective and their task was the same, that is, to watch this disk turn into a green or a red light and to move the right hand up as fast and as accurately as possible as soon as the disk turned to green (Go trials). The right hand had to stay still, resting on the right leg of the participant at all other times, even when the disk turned into a red light (NoGo trials). The green light flashes were also chosen to be much more frequent for the participants to move their hand more often in order to increase the number of visuo-motor correlations and thereby the illusion of ownership in the syncMove condition. During the experiment, participants were expected to keep their body still, relaxed, to refrain from frowning or from producing other facial expressions and to keep their jaw relaxed. Their eyes always had to look in the direction of the disk during each sequence of stimuli, which lasted about 2.5 min.

The disk was flashed for a duration of 50 ms right above the right hand of the avatar. Its diameter was 6 cm. Its color code for the black disk were Red 0, Green 0 and Blue 0; for the red light they were: Red 255, Green 0 and Blue 0; and for the green one: Green 255, Blue 0 and Red 0. It flashed green at 80% of the trials and red at only 20% of the trials. Flashes were 1–1.2 s apart, so the disk was black for 950–1150 ms before the next flash. Each of the three blocks of trials included three sub-blocks, one for each condition (syncMove, noMove and classic), the order of which were counterbalanced across blocks and across participants. There was a total of 400 light flashes, 320 green and 80 red. The disk was black the rest of the times. As mentioned, in the syncMove block, the right hand of the avatar was moving just as the right hand of the participant. Throughout the noMove one, the avatar’s right hand remained immobile. In the classic block, there was no avatar and no virtual room, just red or green flashes in a dark background. After each block, there was a break during which participants were asked to close their eyes and relax as the song “Happy” by Pharell William was played.

After the VR exposure, participants had to answer a brief questionnaire aimed at assessing the degree to which they felt embodied in the avatar, the degree to which they felt located in the virtual room in front of that avatar and the degree to which they felt in the research lab. This is, by mere definition, a fragmentation of the self as it is opposite to the feeling one has to be entirely at a unique location. The self could be fragmented into three pieces: the first in the lab in the real body, the second, in the virtual room in front of the avatar and the third, in the body of the avatar. The questionnaire included four items to be rated on a (0–10) point Likert Scale: the BeingInLab item measured how strongly participants felt they were in the laboratory where we conducted the experiment. It was measured by asking: put a circle to the number corresponding to the degree of feeling of being in the actual lab room where we were sitting when the woman moved her hand like you did (¿Independientemente, pon un circulo al numero correspondiente al grado en el que tu sentias en la sala de laboratorio real donde estabamos sentados cuando la mujer movio la mano como tu lo hiciste?). The PlaceIllusion item measured how strongly participants felt they were in the virtual environment. It was assessed by asking: could you tell us to what degree you felt you were in the virtual environment (¿Podrias decirnos en qué grado sentiste que estabas en el entorno virtual?). The DistractedAvatar item measured how much participants felt distracted by the avatar in front of them. It was assessed by saying: we would like to know the extent to which the woman distracted you during the task (¿Nos gustaria saber el grado en que la mujer te distrajo durante la tarea?). The EmbodiedAvatar item measured how strongly participants felt embodied into the virtual body they saw in front of them. It was assessed by asking: however, independently, make a circle to the number corresponding to the degree to which you felt embodied in the woman when she moved like you did (¿No obstante, de forma independiente, haz un circulo al numero correspondiente al grado en el que tu sentiste encarnado en la mujer cuando ella se movia como tu lo hiciste?). These latter two questions were only asked after the syncMove and the noMove condition-block.

### Data Acquisition

Hand tracking was sampled at 255 Hz. Each trial had a bit more than 255 amplitudes. We measured the maxima of these amplitudes for each trial, each condition and each subject.

The VR application and the electroencephalogram (EEG) data acquisition were carried out on two separate PCs the clocks of which were synchronized *via* the network time protocol (NTP). The EEG was recorded with Ag/AgCl electrodes mounted in an elastic cap from Easycap at 13 of the sites of the extended International 10–20 system (Sharbrough et al., 1991), which were selected to capture the P300b. FCz, Cz and Pz CP1/2, PO3/4, C3/4, P3/4, CP5/6 sites were thus used. The reference electrode was placed on the right earlobe and the EEG was amplified by g.USBamps (g.tec medical engineering GmbH, Schiedlberg, Austria). The half amplitudes cut-offs of their high- and low-pass filters were set at 0.1 and 30 Hz, respectively, with an additional electronic notch filter to remove 50 Hz contamination. Signals were then digitized on-line at a sampling rate of 255 Hz and output into a computer using the MATLAB R2013a Simulink software (The MathWorks, Inc., Natick, MA, USA).

### Data Processing, Measures and Analyses

Reaction times (RTs) of the correct responses of the Go trials were not computed because hand lifts were of different amplitudes and of different speeds across trials and participants. It was too difficult to decide when a hand lift was complete enough to measure the time it took to reach that completeness. In any case, only the oddballs, that is, the NoGo trials, were relevant for the testing of the hypothesis, as they were the trials on which we measured our P300b effect. For these trials, motor responses are errors. Such errors are frequent since Go trials were so much more frequent than NoGo trials. In Go/NoGo protocols, such errors rates provide an estimate of accuracies. In our protocol, all the participants made such errors. We calculated them by measuring first, all the maximal heights that were reached by each participant at each of the trials of NoGo conditions. We computed the mean of these maxima. We then counted the number of NoGo trials where hand lifts were above that mean in each of the three NoGo conditions. Finally, we divided that last number by the total number of trials of each of these conditions.

EEG offline analysis was performed with MATLAB R2013a using the BBCI (Blankertz et al., 2016) and EEGLAB (Delorme and Makeig, 2004) toolboxes. The continuous EEG signal was cut into epochs corresponding to each change of the disk starting 200 ms before and ending 800 ms after the onset of this change. To place these epochs on the baseline, their mean voltages within the −200 to 0 ms time window were computed and subtracted to each point of the entire epoch. Artifacts were identified as voltages having a maximum absolute amplitude higher than 75 μV. When only one channel in an epoch had such artifacts, this channel was recomputed by interpolation of surrounding channels using the “pop_interp” function of the EEGLAB toolbox. When more than one channel included such artifacts, the whole epoch was excluded. The mean number of excluded epochs was 8.0 (*STD* = 13.7). The remaining EEG epochs of frequent-Go and oddball-NoGo trials were averaged separately for the syncMove-, the noMove- and the classic-conditions, leading to six ERPs for each participant.

P3b amplitudes were measured at electrode site Pz, where the P300b effect is usually the largest. Note that, before these particular measures, the continuous EEG signal was smoothed with a 2nd order Butterworth filter between 0.1 Hz and 10 Hz before cutting it into epochs and setting it to the baseline as mentioned above. The mid-latency (ML) method (Guillem et al., 2001) was then used to determine the time window of P300b measure in the following way. The difference curve between the grand averages of the Go and the NoGo trials for all three conditions was computed first. The latency of the peak of the P300b effect (P3b) was then measured, as well as the ML between the N2 effect and this P3b in order to compute the delay (t) between these two latencies (t = P3b − ML). The mean voltage of the ERPs of each participant in each condition was then computed in the (P3b ± t) time window.

In order to control for the allocation of attentional resources, the amplitudes of the P1 and N1 ERPs were measured at electrode sites PO3 and PO4. This time, since these ERPs includes much higher frequencies than the P3b, no additional filter was used before isolating the EEG epochs. The grand averages ERPs of all conditions were used to define the time windows of measures of the mean voltage amplitude of the P1s and N1s. These windows were computed using the ML between P1 and N1 peaks in the same way as described above for the N2-P3b.

All statistics on these measures were performed with Stata 13 (StataCorp LP, College Station, TX, USA). Since each participant carried out all three experimental conditions, a mixed-effects model was used with fixed effects “condition” and random effects over the “individual participants.” For P300b amplitudes we used a Multilevel Mixed-Effects Linear Regression (the “mixed” function in Stata) followed by *post hoc* paired comparisons, which were corrected with Scheffés criterion for multiple comparisons. Normal distributions of residuals were tested with the Shapiro–Wilk test. A Multilevel Mixed-Effects Ordered Logistic Regression (the “meologit” function in Stata) was used to analyze the questionnaire data, given their ordinal nature. The relationship between EmbodiedAvatar and P3b amplitudes was analyzed using a mixed effects regression (the “mixed” function in Stata), with fixed effects over the factor condition, covariate EmbodiedAvatar and random effects over the individuals.

## Results

### Analyses of Questionnaire Responses

**Figure 2:**
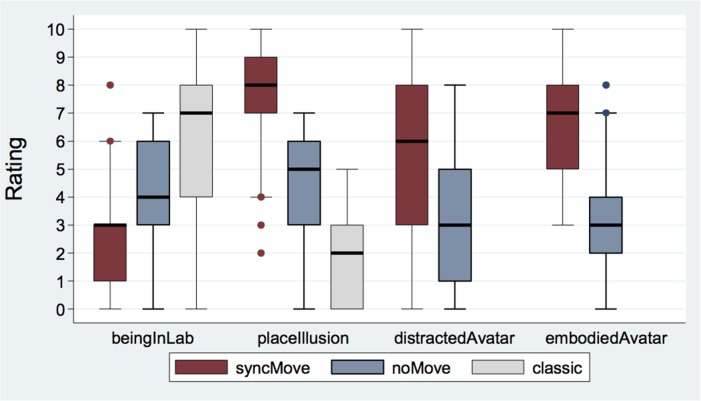
Boxplot of questionnaire responses for the three different conditions syncMove, noMove, and classic. Note that questionnaire responses for distractAvatar and embodiedAvatar were only possible after syncMove and noMove conditions because there was no avatar in the classic condition (*N* = 27).

Figure 2 displays box plots of the mean scores for the different questionnaires. The smallest feeling of BeingInLab was found in the syncMove condition, the more intense, in the classic condition while that in the noMove control condition was intermediate (*z* = 6.24, *P* < 0.001). *Post hoc* paired comparisons showed that the classic (*Median* = 7, *IQR* = 4) had higher ratings than the noMove (*Median* = 4, *IQR* = 3, *z* = 4.42, *P* < 0.001) and than the syncMove condition (*Median* = 3, *IQR* = 2; *z* = 6.24, *P* < 0.001). The noMove- had higher ratings than syncMove-condition (*z* = 4.02, *P* < 0.001).

The PlaceIllusion item measured how strongly participants felt they were in the virtual room. Strongest in the syncMove condition, this feeling was of intermediate intensity in the noMove and minimal in the classic conditions (*z* = −6.80, *P* < 0.001). *Post hoc* paired comparisons showed lower ratings for the classic- (*Median* = 2, *IQR* = 3) than the noMove- (*Median* = 5, *IQR* = 3; *z* = −4.53, *P* < 0.001) and the syncMove-condition (*Median* = 8, *IQR* = 2; *z* = −6.80, *P* < 0.001). The noMove-condition has also lower ratings than the syncMove one (*z* = −5.63, *P* < 0.001).

The mixed logistic regression revealed significant differences between the degrees to which participant felt distracted by the avatar across conditions (*z* = −3.23, *P* = 0.001). This distraction was lower in the noMove- (*Median* = 3, *IQR* = 4) than in the syncMove- (*Median* = 6, *IQR* = 5) condition.

The same type of regression showed a large effect of condition on the extent to which participants felt embodied in the avatar (*z* = −4.97, *P* < 0.001). Participants gave higher EmbodiedAvatar ratings during the syncMove (*Median* = 7, *IQR* = 3) than during the noMove (*Median* = 3, *IQR* = 2) condition (*z* = −4.97, *P* < 0.001).

### Behavioral Data

The average error rate for the SyncMove condition was 25.42%, that for the noMove was 26.98% and that for the classic was 27.70%. There was no significant difference between these mean error rates (standard deviations were around 10%).

### Electrophysiological Results

**Figure 3:**
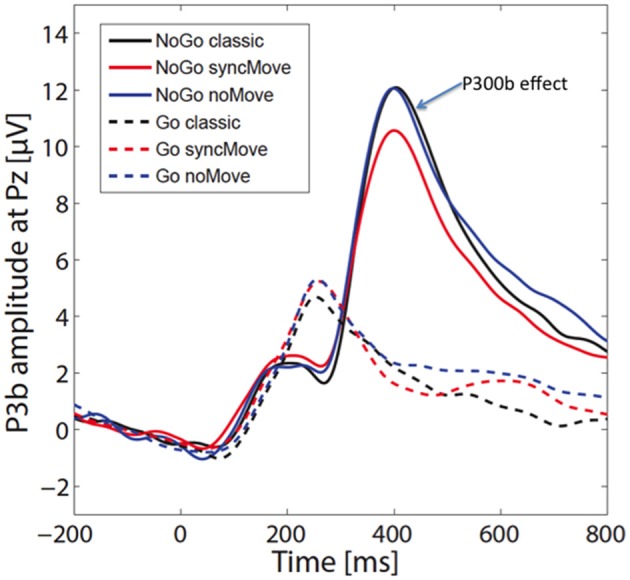
Grand averages (*n* = 27) of the event-related brain potentials (ERPs) at electrode Pz for the six different conditions. The 0 indicates the time point when the visual stimulus was delivered. The blue arrow points to the P300b effect. The vertical scale is in microvolts.

**Figure 4:**
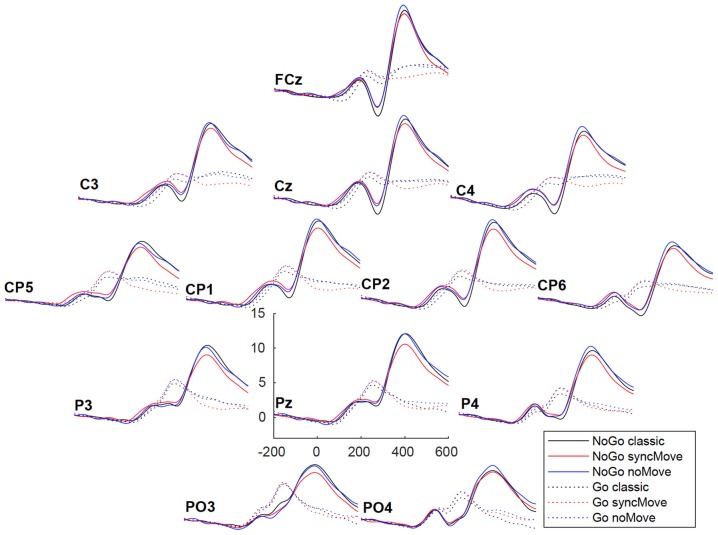
Grand averages (*n* = 27) of the ERPs at all electrodes for the six conditions. The vertical scale (*y* axis) is in microvolts.

**Figure 5:**
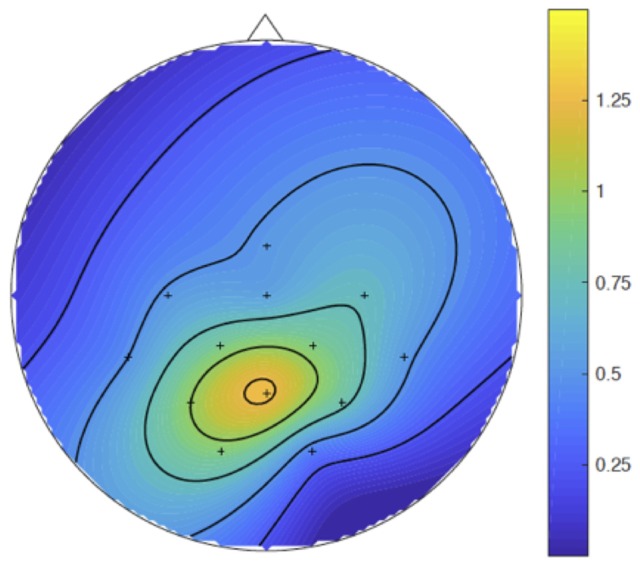
Parietal maximum of the P300b effect. Spline inter- and extra-polated isovoltage maps made, in the P300b time windows, with the results of the subtraction of the mean voltages of the grand averages of the syncMove condition from the mean voltages of the grand averages of the noMove condition. The color scale is in microvolts.

Figure 3 shows the ERPs. The amplitudes of the P300b appear smaller in the critical syncMove condition than in the noMove control- and the classic-condition. These differences were maximal at the parietal site (Pz, Figures 4, 5). The mixed effects ANOVA showed that this difference was significant, (*z* = 3.36, *P* = 0.001). *Post hoc* paired comparisons revealed that P300b amplitudes in the syncMove condition (*Mean* = 9.33) were lower than in the noMove condition (*Mean* = 10.58, *z* = 3.18, *P* = 0.006) and in the classic condition (*Mean* = 10.66, *z* = 3.36, *P* = 0.004) and that there was no difference between the noMove and the classic condition (*z* = 0.18, n.s.).

Our data did not show a relationship between P3b and embodiedAvatar (mixed effects regression, *z* = 0.23, n.s.) nor a relationship between P3b and distractedAvatar (mixed effects regression, *z* = –0.40, n.s.).

N1 amplitudes did not differ across conditions, neither at PO3 (mixed effects ANOVA: *z* = −0.75, n.s.) nor at PO4 (mixed effects ANOVA: *z* = –0.85, n.s.). Similarly, P1 amplitudes were not different among conditions, neither at PO3 (mixed effects ANOVA: *z* = 0.84, n.s.) nor at PO4 (mixed effects ANOVA: *z* = –0.24, n.s.).

## Discussion

We thus tested whether the amplitudes of the P300b ERP would be smaller in case the stimulus eliciting this ERP could be bound to only a fragment of the self of participants rather than to their whole self. This restricted binding was made possible by embodying our participants in an avatar who was in a virtual room and by presenting stimuli there. These stimuli thus had to be bound to that fragment of the self rather that to the one corresponding to the real participant in the real lab room. An experimental setting where participants were immersed in a virtual room in front of an avatar who could move like they did was thus used. The ratings of the participants at the debriefing session confirmed that the syncMove condition of the setting, namely the one where the avatar was moving like participants were, was efficient at inducing this fragmentation. Participants felt more embodied in the avatar than they did in the control condition. It also showed that they felt more in the virtual room than in the lab in this syncMove- than in the noMove-condition. As expected, P300b were of smaller amplitudes in the syncMove- than in the noMove- and in the classic-condition. We then verified that these smaller P300bs could not be due to the fact that participants may have been more distracted by the avatar in the critical condition than in the other conditions by checking the absence of correlation with the distraction scores and by verifying that well-known ERP indexes of attention, that is, P1s and N1s (Hillyard et al., 1995; Hillyard and Anllo-Vento, 1998), were as large in the critical condition as in the control and classic condition. This absence of attention bias is further supported by the fact that the error rates of the noGo trials were not significantly different across the three conditions. Results thus support the hypothesis that the drastic reduction of P300b amplitude found in schizophrenia could be due to their particular self-representations, which could also explain why, when they get better, their P300b partially normalizes (e.g., Coburn et al., 1998).

As mentioned, the reduction of P300b amplitude would be due to the fact that stimuli occurred only in the VR room. Thus, they could *only* be bound to the fragment of the self that was in the virtual room facing the avatar and/or to the fragment that was in the body of the avatar. They could not be bound to the fragment of the self that was in the lab, as participants knew that no stimuli appeared in the lab room. P300b would be maximal only when the self is united and stimuli bound to the whole self. Similarly, in schizophrenia patients who are going through a classical oddball experiment, stimuli would be bound only to the fragment of the self that is in the real lab. They would not necessarily be bound to fragments of the self that correspond to delusional identities, for instance, and are not part of the real lab. Similarly, they would not necessarily be bound to the parts of the self that correspond to the voices or to the person(s) they feel in their mind etc.

The absence of negative correlation between the P300b amplitude of participants and the precise degree to which they felt embodied in the avatar is at first a bit puzzling. It could be due to two things. First, to the fact that, to be reliable, between-subject statistical analyses require many more participants when the dependent variable is an ERP. Too many factors, other than fragmentation, may be at stake in P300b amplitude, such as the thickness of the bones of head of the participants, as well as that of their scalp and hair, the particular orientation of their dipoles etc. Second, this absence of correlation could be due to the theoretical impossibility to *accurately* estimate the degree to which one felt in the lab, or in the avatar. In effect, this accuracy depends on a recall of the experience of fragmentation after the test, and, therefore, when there is no more VR and when the normal participant is fully “re-united” in his/her own body in the real room.^2^^,^^3^ It thus makes much more sense to use the raw evaluations showing that, in the condition where the avatar moved like the participants, they reported an embodiment that was on average greater than that of the control conditions (i.e., of the noMove and of the classic condition), a fact that has already been demonstrated in prior studies (Sanchez-Vives et al., 2010; Slater et al., 2010).

Before discussing the possible impacts of the results on the functional significance of the P300b, it may be useful to remind that this ERP have also been called the P3 because it is the third sizeable deflection of the ERPs. The “b” in the terms P3b and P300b came later, once another third deflection, the P3a, was found to be maximum a bit earlier, over frontal sites. The original “P300,” the P3, the P3b and the P300b are thus terms that designate the same thing. The relation hypothesized here, between the P300b and the representation(s) of the self, fits literature data. In effect, this late, posterior and positive ERP is an index of the consciousness of the meaning of any stimulus/event in any cognitive task, even when the stimulus is in fact the meaningful *omission* of the predicted stimulus (Tarkka and Stokic, 1998). The relation between the P300b and the self fits the fact that the consciousness of this meaning is always associated to the self in a sensation that can be verbalized as: “*I* am currently perceiving the stimulus-event for which *I* have been asked to provide this response.” This should be true not only for the classical P300 potential, which was called this way only because it was originally discovered in a protocol where it peaked around 300 ms after the onset of the unexpected stimuli used (Sutton et al., 1965). It should be true also for other LPPs, which appear to belong to the same family of ERPs the latency of which just vary according to the nature of the stimulus and to the difficulty of the cognitive task participants have to perform. For instance, for word stimuli, it usually peaks around 600 ms (Kutas et al., 1977) and is therefore sometimes called P600 (e.g., van Herten et al., 2005). The more difficult the cognitive task is, the later will be the peak, even for simple stimuli (Polich, 1987). The potential has also been called the late positive component (LPC, e.g., Juottonen et al., 1996) or, as mentioned, the late posterior positivity (LPP, e.g., Schupp et al., 2004), a phrase that captures both its electrical polarity, the parietal location of its maximum on the scalp and its delayed occurrence relative to earlier ERPs.

The relation between LPPs and the representation(s) of the self also fits ERP results pertaining to the *conscious* appraisal of the meaning of the triggering event in the cognitive task at stake. For example, when participants have to privilege the accuracy of their judgment over its speed, and thus, most likely, when they provide their answers only once they become fully conscious of the meaning of the stimulus for the task, the latency of the LPP peak correlates with RTs (Kutas et al., 1977). Further evidence on the relationship between the LPP and consciousness comes from studies of the attentional blink, which show that only consciously perceived stimuli elicit LPPs (Luck et al., 1996; Vogel et al., 1998; Sergent et al., 2005, for a recent review of ERPs and consciousness, see also Rutiku and Bachmann, 2017). The relationship between P300b and the consciousness of the meaning of an event is also corroborated by the location of several generators of this ERP (e.g., Bledowski et al., 2004; Di Rollo et al., 2016), which are in brain structures that are part of neuronal networks assumed to underlie consciousness (Demertzi et al., 2013). It has to be noted, though, that this relation between the LPP and consciousness pertains, first, to the meaning of the event and not necessarily to the conscious perception of the stimulus itself (Koch et al., 2016). In effect, as previously mentioned, an LPP is also observed in response to the meaningful omission of a predicted stimulus (e.g., Tarkka and Stokic, 1998). That the location of the maximum of the P300b observed on the scalp around parietal sites does not depend to a large extent on the modality of the stimulus (auditory or visual) used (e.g., Katayama and Polich, 1999) is yet another indication that its main determinants are not the conscious perception of the *physical* stimulus itself.

Additionally, the scalp distribution of the P300b fits the coding of the meaning of the event in the task for the *participants themselves*. In effect, parts of its cortical generators are parietal suggesting that some of these dorsal stream areas code the new event with egocentric values. As mentioned, it also fits the fact that when we are conscious of something, like the meaning of an event, this consciousness is bound to the self. And on the other hand, the consciousness of the fact that we have just been conscious of something, namely metacognition, seems to be indexed by the slow positive waves that sometimes follow the P300b (Müller et al., 2016).

At the opposite, the perception of the physical features of the stimulus (e.g., is it a red disk) and of its nature (i.e., it is the image of a red ball), could be indexed by the P1 and by components of the P2, respectively, given that these ERPs may reflect forms of consciousness that cannot be reported afterwards, such as iconic memory, which do not systematically translate into working memory. In effect, this latter translation seems to be specific to the P300b (Donchin and Coles, 1988). In contrast, N1, N300 and N400 could index various types of inhibitions (for N1s see Touzel et al., 2018; for N2s, see Bruin and Wijers, 2002; Jodo and Kayama, 1992; and Roche et al., 2005; for N300s, see Debruille et al., 2012 and for N400s, see Debruille, 2007; Debruille et al., 2008 and Shang and Debruille, 2013).

Finally, it seems logical to see the binding of the representations of the self with representations of the stimulus, such as those of its meaning in the task, as a condition for the successful encoding in episodic memory. Such encoding precisely appears to be indexed by one of the components of the LPP, which makes its total amplitude greater (Paller et al., 1987). These facts may be important for schizophrenia patients who suffer from a deficit in episodic memory (Aleman et al., 1999) that has strong detrimental consequences on their functional outcome. Their self fragmentation might also help to understand the mechanisms of their episodic memory deficit.

For all these reasons, we thus hope that this work will trigger new explorations of the P300b to further examine its systematic relation with the self and consciousness whatever the cognitive task at hand. On the other hand, we also hope that it will stimulate the use of EIVR in schizophrenia patients. In effect, this use might help some of these patients to understand their self fragmentation. Eventually, these patients may then be more capable of integrating some of the fragments of their selves into a more global representation. They would then do like the participants of our experiment who knew that their embodiment in the avatar of the virtual room and their embodiment in their real body in the lab room could be both integrated in a single representation: that of being a participant of a VR experiment. The P300b reduction of patients would then become small, such as the one of the subjects of our experiment in the syncMove condition.

## Author Contributions

BS developed the experimental design together with JD. He implemented all prior pilot versions of the protocol as well as the final one. Together with JD or MH, BS ran all the participants, processed the EEG, wrote part of the “Materials and Methods” section of the article that pertains to VR and stimulus presentation. BN taught JD and MH how to test subjects in the Event-Lab. BN processed the behavioral data and the EEG, computed the ERPs, measured their mean voltage in selected time windows, ran the statistics and wrote the “Results” section. MH tested half of the participants with BS. JD had the initial idea to use VR to induce fragmentation of the self. Participants were payed on his fund. He tested half of them with BS and wrote the initial version of the article. All authors corrected several versions of the article and agreed on its final content. The experiment was run in the Event-Lab supervised by Mel Slater (MS) and Mavi Sanchez-Vives. MS had many key insights for the construction of the experimental design.

## Conflict of Interest Statement

The authors declare that the research was conducted in the absence of any commercial or financial relationships that could be construed as a potential conflict of interest.

## Footnotes

^1^On the other hand, the so-called “negative symptoms” of schizophrenia (e.g., lack of motivation, blunted affect‥.) that are typical of the residual forms, go with a poverty of thoughts, which, most likely, extends to the representations of the self. These patients are sometimes nicknamed “zombies” as the lack of personality is sometimes blatant.

^2^One could then argue that the embodiment-in-avatar question should have been asked verbally, during the blocks. But this question would have been bound to the real self in the lab room and would have decreased the embodiment in the avatar.

^3^This impossibility could be similar to the one schizophrenia patients in remission experience when trying to narrate what they went through during their psychotic episode.
